# A systematic review of correlates of sedentary behaviour in adults aged 18–65 years: a socio-ecological approach

**DOI:** 10.1186/s12889-016-2841-3

**Published:** 2016-02-17

**Authors:** Grainne O’Donoghue, Camille Perchoux, Keitly Mensah, Jeroen Lakerveld, Hidde van der Ploeg, Claire Bernaards, Sebastien F. M. Chastin, Chantal Simon, Donal O’Gorman, Julie-Anne Nazare, on behalf of the DEDIPAC consortium

**Affiliations:** Centre for Preventive Medicine, School of Health & Human Performance, Dublin City University, Dublin 9, Republic of Ireland; CarMeN Laboratory, INSERM U1060, Lyon 1 University, CRNH-Rhône-Alpes, CENS, Lyon, France; EMGO Institute for Health and Care Research, VU University Medical Centre, Amsterdam, Netherlands; TNO, Leiden, The Netherlands; Institute of Applied Health Research, School of Health and Life Science, Glasgow Caledonian University, Glasgow, UK

**Keywords:** Sitting, Sedentary behaviour, Determinants, Correlates, Adults, Ecological model, Intrapersonal, Interpersonal, Environment, Policy-related

## Abstract

**Background:**

Recent research shows that sedentary behaviour is associated with adverse cardio-metabolic consequences even among those considered sufficiently physically active. In order to successfully develop interventions to address this unhealthy behaviour, factors that influence sedentariness need to be identified and fully understood. The aim of this review is to identify individual, social, environmental, and policy-related determinants or correlates of sedentary behaviours among adults aged 18–65 years.

**Methods:**

PubMed, Embase, CINAHL, PsycINFO and Web of Science were searched for articles published between January 2000 and September 2015. The search strategy was based on four key elements and their synonyms: (a) sedentary behaviour (b) correlates (c) types of sedentary behaviours (d) types of correlates. Articles were included if information relating to sedentary behaviour in adults (18–65 years) was reported. Studies on samples selected by disease were excluded. The full protocol is available from PROSPERO (PROSPERO 2014:CRD42014009823).

**Results:**

74 original studies were identified out of 4041: 71 observational, two qualitative and one experimental study. Sedentary behaviour was primarily measured as self-reported screen leisure time and total sitting time. In 15 studies, objectively measured total sedentary time was reported: accelerometry (*n* = 14) and heart rate (*n* = 1). Individual level factors such as age, physical activity levels, body mass index, socio-economic status and mood were all significantly correlated with sedentariness. A trend towards increased amounts of leisure screen time was identified in those married or cohabiting while having children resulted in less total sitting time. Several environmental correlates were identified including proximity of green space, neighbourhood walkability and safety and weather.

**Conclusions:**

Results provide further evidence relating to several already recognised individual level factors and preliminary evidence relating to social and environmental factors that should be further investigated. Most studies relied upon cross-sectional design limiting causal inference and the heterogeneity of the sedentary measures prevented direct comparison of findings. Future research necessitates longitudinal study designs, exploration of policy-related factors, further exploration of environmental factors, analysis of inter-relationships between identified factors and better classification of sedentary behaviour domains.

**Electronic supplementary material:**

The online version of this article (doi:10.1186/s12889-016-2841-3) contains supplementary material, which is available to authorized users.

## Background

The time that adults spend sedentary or put simply doing “too much sitting” has recently been proposed as a population wide issue that has deleterious effects on health outcomes. New evidence links excessive sitting in adults with lifestyle related diseases such as obesity, type II diabetes, cardiovascular diseases, pulmonary disease and cancer [1–3]. It has been shown that sedentary time has specific metabolic consequences even among those meeting the moderate-to-vigorous physical activity guidelines. A gradient exists with higher morbidity and mortality rates among those who spend more of their time being sedentary, independent of whether or not they engage in regular physical activity [2, 3]. Typically, “sedentary” is defined as any waking activity that requires an energy expenditure ranging from 1.0 to 1.5 (basal metabolic rate) while in a sitting or reclining posture [4, 5].

The focus to date on factors that influence sedentary behaviours has mostly been on individual level factors such as biological, psychological and behavioural [1, 6]. However, it has become apparent that these are not stand-alone factors and addressing them in isolation will not result in a significant change in sedentary behaviours [1]. Social, environmental and policy factors may also need to be taken into account. The current rationale is that factors that influence sedentary behaviour can be conceptualised using models such as the socio-ecological model [6]. The socio-ecological approach emphasises that focus should not only be on individual behavioural factors but also on the multiple-level factors that influence the specific behaviour in question [7], thus focusing on the interrelationships between individuals and the social, physical and policy environment. This model places the individual within an ecosystem that acknowledges individual behaviour is dependent on the dynamic relationships between it and other determinants or correlates relating to the environment, economy, political and social agendas [7]. The model has been widely applied to research looking at what influences physical activity behaviours [7] and it has been suggested that a comprehensive approach, such as that offered by the socio-ecological model is essential for examining the multiple level factors that might determine sedentary behaviours [1]. This ecological model provides a framework that facilitates mapping the multiple domains of sedentary behaviour, while at the same time assuming multiple levels of influence [1].

A previous review investigating sedentary behaviour correlates in adults identified numerous intrapersonal factors relating to sedentary behaviour, several which are non-modifiable (for example, age and gender) [6]. However they did not identify many factors or correlates outside of the individual. Potentially significant factors such as the built, physical, social and policy environments need to be identified and since the publication of that review there have been several studies investigating the environmental influences on sedentary behaviours, both at an individual and community level [8–18]. These factors need systematic identification so that they can be considered along with individual level and social correlates in the development of interventions to address sedentary behaviours. Therefore, the aim of this study is to comprehensively review the quantitative (observational and experimental) and qualitative literature on determinants and correlates of sedentary behaviours in adults aged between 18 and 65 years. The overall objectives are to (i) provide an update on previously reported factors, (ii) identify novel intrapersonal (individual), interpersonal (social), environment and policy factors, (iii) investigate the interactions between the different factors, (iv) identify gaps in the existing literature and (v) provide recommendations for future research in this area.

## Methods

This systematic review is one of three reviews, part of the Joint Programming Initiative’s funded Determinants of Diet and Physical Activity (DEDIPAC) consortium [19] aimed at reviewing and updating the current evidence base on the determinants and correlates of sedentary behaviour across the life course, with two other reviews focusing on children and adolescents (<18 years) and older adults (>65 years). A common protocol for the three DEDIPAC systematic reviews across the life course was developed and is available from PROSPERO (PROSPERO 2014:CRD42014009823).

### Search strategy

Five electronic databases (PubMed, Embase, CINAHL with full text, PsycINFO and Web of Science) were searched. The search strategy was based on four key elements: (a) sedentary behaviour and its synonyms (e.g., sedentariness); (b) correlates or determinants and its synonyms (e.g., correlates, factors); (c) types of sedentary behaviour (e.g., TV viewing, gaming) and (d) possible correlates or determinants of sedentary behaviour (e.g., environmental, behavioural and socio-demographic). Terms referring to these four elements were used as MESH-headings and title or abstract words in all databases. A complete list of the search terms is available in the additional materials section (Additional file 1: Table S1). In addition to the above, the reference lists of all included articles were scanned for articles that met the inclusion criteria. Any retrieved articles underwent the same selection process as the other articles.

### Inclusion criteria

Scientific peer reviewed published papers written in English from January 2000–September 2015 were included in the review (conference abstracts, reports and thesis were excluded). Adults were defined as any population aged ≥18 years and <65 years. Articles whose primary outcome focuses on specific patient groups/pathology were excluded. Study designs eligible for inclusion were observational studies (cross sectional, case control and prospective), experimental studies (randomised controlled trials, quasi-experimental trials) and qualitative studies. In terms of sedentary behaviour outcome measures, one or more of the following were acceptable; total sedentary or sitting time (e.g., minutes per day) or time spent in one or more of the following specific domains of sedentary behaviour; time spent watching TV, screen time (in any domain i.e., leisure or work), occupational sitting time or transport related sitting time. Both objective and subjective measurement outcomes were included (cut off point for accelerometric sedentary behaviour = ≤100 counts per minute).

### Selection process

The selection process consisted of three phases. In the initial phase, two reviewers (GO’D and KM) independently screened the yielded articles based on title. In the case of doubt, the articles were included in the abstract review phase. In phase two, all articles selected from the initial phase had their abstract reviewed and assessed by three independent reviewers (GO’D, JAN and CP). Any disagreement was resolved by the third reviewers (JL, HvdP and CB). In the final phase, the remaining articles were fully reviewed by the same three independent reviewers using the pre-determined inclusion criteria. Any disagreement between reviewers in this phase was resolved by discussion within the wider team.

### Data extraction

An eight item standardised pre-piloted data extraction form was used to extract data from the included studies under the following headings: (i) general information; (ii) sample characteristics; (iii) study design; (iv) measurement of sedentary behaviour; (v) measures of factors that influence sedentary behaviour; (vi) statistical analyses; (vii) results reported and (viii) general conclusions. Additional file 1: Table S2 (additional files) provides further detail.

### Risk of bias

To assess the risk of bias, the quality assessment tool ‘QUALSYST’ from the “Standard Quality Assessment Criteria for Evaluating Primary Research Papers from a Variety of Fields” (Alberta Heritage Foundation for Medical Research) was used. This pragmatic tool incorporates two scoring systems, allowing quality assessment to be conducted on both quantitative and qualitative research [20]. As both quantitative and qualitative study designs were included in this review, this tool was deemed appropriate (Additional file 1: Tables S3 and S4). Fourteen items for each quantitative study and 10 for each qualitative study were scored depending on the degree to which the specific criteria were met or reported (‘yes’ =2, ‘partial’ =1, ‘no’ =0). Items not applicable to a particular study design were marked ‘n/a’ and excluded when calculating the summary score. The three reviewers involved in article selection assessed quality independently (GO’D, JAN and CP). All articles were reviewed by at least two of the three reviewers. A quality assurance process enabled cross checking of quality assessment. Discrepancies were resolved through discussion.

### Data synthesis

A narrative synthesis of the findings of the review is provided structured around the ecological model of sedentary behaviour [1]. A narrative synthesis was conducted because of the high levels of clinical, methodological and statistical heterogeneity, making data pooling inappropriate. Qualitative tables illustrate the main study characteristics and show the individual, social and environmental factors that have been investigated and their relationships to sedentary behaviour. Direction and strength of the association between these factors and the different categories of sedentary behaviour are summarised, as well as the gender categories under study. A thematic synthesis was used to summarise the qualitative studies and the findings are integrated with the quantitative findings using the parallel synthesis approach recommended for mixed-methods research synthesis [21].

## Results

**Fig. 1:**
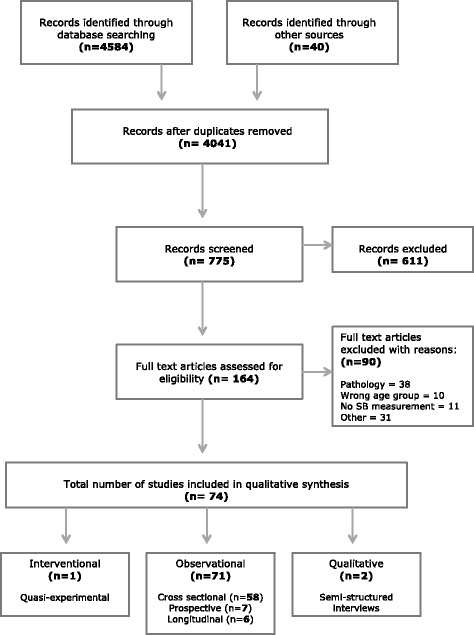
PRISMA diagram of study selection process

The process for undergoing the literature search and screening, including numbers of papers excluded and reasons for exclusion is illustrated in Fig. 1. In summary, the electronic search yielded 4584 records and a manual search of personal databases and recent publication reference lists yielded a further 40 records, resulting in a total of 4624 records. 583 duplicates were removed. Of the remaining 4041 records, 3967 were excluded throughout the screening process. Overall, 74 papers passed the eligibility criteria to be included in the review.

### Study characteristics

Table 1 provides a detailed overview of all the included study characteristics. Of the 74 studies included, 21 were conducted in North America, 23 in Europe, 24 in Australia, one in New Zealand and one across three continents (United States, Australia and Belgium). The remaining four were conducted in Asia. All studies apart from three (one experimental and two qualitative) were observational. The most common observational study design identified was cross sectional (*n* = 58). Participant sample sizes ranged from 10 to 246,920 adults with age ranging from 18 to 65 years in all studies but one where the age ranged from 16 to 96 years [22]. In terms of gender, eleven studies were based on women only and two on men while the remainder included both. Participants from a broad range of socio-economic backgrounds were included across the various studies with only eight addressing specific working groups.

**Table 1:** Overview of study characteristics

Author^REF^	Number, age, gender	Design	Outcome	Individual factors	Environmental factors	Interpersonal factors	Quality score
Astell-Burt [14]	246920 adults 74–106 years 48 % men	CS	Sitting time		Proximity of green spaces		0.86
Ballard [35]	116 men Mean age = 19.54	CS	TV Viewing Video games Reading	BMI, body fat %, frequency of exercise, length of exercise, days of moderate activity, days of walking			0.86
Barnett [73]	3334 adults 45–79 years 48 % men	PO	Changes in TV viewing time	Age, retirement, social class, levels of PA			0.90
Bowman [32]	9157 ≥20 years	CS	TV Viewing	Age, sex, education, race, ethnicity			0.86
Chau [38]	10785 adults 15–69 years 42 % men	CS	Leisure sitting time Sitting time at work	Occupational activity			0.90
Clark [68]	Young cohort: *n* = 5215, age 24.6 (1.5) mid-aged cohort: *n* = 6973, age 52.5 (1.4) 100 % women	P	Hours per day total sitting (visiting friends, reading, driving, reading, watching TV or working at desk/computer) on week and weekend days	Life events in the previous 12 months: major illness surgery, return to study, moving out, decreased income, menopause,		Life events in the previous 12 months: decline health of close family, birth of child, begin work, loss of job, change at work, divorce, new relationship, retirement, spouse retirement, child leaving home	0.84
Clark [48]	10951 adults 25–91 years 45 % men	CS	Time spent in TVSE	Age, education, household income, employment status	Living outside the state capital city	Living arrangements	0.95
Clemes [39]	170 adults 18–65 years 30 % men	CS	Time spent sedentary^O^	Levels of PA outside work		Workdays vs. non-workdays	0.77
Coogan [8]	59000 women 21–69 years	CS	TV Viewing		Neighbourhood walkability, neighbourhood SES		0.81
Conroy [45]	128 adults Mean age = 31,3 (SD = 1,1) 41 % men	CS	Time spent sedentary^O^	Sedentary habits, daily intentions to limit sedentary behaviour, levels of PA			0.86
Crespo [86]^a^	1313 adults Mean age = 45 (SD = 10) 56 % men	CS	Time spent sedentary^O^	Age, gender, education, ethnicity	Worksite promotion index including: shower facilities at work, lockers for clothes at work, safe bicycle storage		0.95
De Cocker [64]	5562 women	L	Changes in sitting time	Weight			0.91
De Cocker [54]	993 adults mean age 51	CS	Occupational sitting time	Gender, age, educational level, household income, self-reported health, self-efficacy about sitting less, intention to sit less		Social norm towards sitting less in work, social support towards sitting less in work	0.91
De Wit [57]	3005 adults 18–65 years 34 % men	CS	Time spent watching TV or using PC	Depressive symptoms, anxiety disorders			0.82
Den Hoed [69]	1654 adults twins 2 % men Mean age = 56 (SD = 10)	CS	Time spent sedentary^O^	Heritability (additive genetic factors)			0.90
Ding [9]	551 adults 20–70 years olds 39 % men	L	Changes in TV viewing time	Age, gender, education, annual household income, employment status, occupational PA, domestic PA, transport PA	Neighbourhood walkability index, neighbourhood pedestrian infrastructures, aesthetics, traffic-related safety, crime-related safety Neighbourhood SES	Living arrangements, number of children (<18 years) in the household Social interactions and social cohesion, sense of community	0.91
Ding [26]	37570 adults average age 61 year, 54 % female	CS	Time spent driving (motorised transport)	Smoking, alcohol consumption, dietary risk, physical activity levels, sleep quality, BMI, quality of life, self-rated health			0.91
Ekelund [56]	393 adults Mean age = 49,7 (SD = 8) 45 % men	P	Time spent sedentary^O^	BMI, fat mass, waist circumference			0.91
Evenson [70]	359 women ≥16 years	CS	Time spent sedentary^O^	Pregnancy			0.91
Fields [11]	189 adults Mean age = 32 (SD = 10,2) 31 % men	CS	Time spent sedentary outside of work		Residential density, bike facilities, sidewalk, proximity of a bus stop, access to services, recreation facilities, traffic safety, safe park, crime safety		0.82
Frank [83]	10876 adults 46 % male	CS	Car time as passenger or driver		Land use mix, intersection, density, residential density		0.86
George [67]	15 men 35–64 years	Q	Barriers to decreasing sedentary time	Health status and working hours	Weather as a barrier, access to recreation facilities	Social interactions and sense of community and family support	0.82
Granner [31]	189 women 18–60 years	CS	TV viewing Sitting time Time spent sedentary	Age, education, employment status, ethnicity, eat meals or snacks while watching TV, BMI, self-rated health, number of days per month depressed, number of days per month anxious			0.86
Grothe [30]	39 women ≥18 years	CS	Time spent sedentary^O^ TV Viewing Video games Computer work Paper work Phone use Reading Doing artwork Transportation sitting time	Age, education, income, ethnicity, food cravings, BMI, illness			0.90
Hadgraft [40]	1235 adults mean age 53.7 38 % women	CS	Occupational sitting time and TV viewing time	Income, profession, energy intake, educational attainment, leisure time physical activity, BMI		Marital status	0.90
Hagströmer [13]	1172 adults 19–69 years 45 % men	CS	Time spent sedentary^O^		Region, season		0.81
Hamer [29]	3923 adults Mean age = 51 (SD = 15,8)	CS	Time spent in TVSE	Deprivation, BMI, mental health, physical function, psychological distress, smoking, alcohol intake, fruits and vegetables intake			0.86
Hamrik [50]	19-90 years	CS	Time spent sedentary	Age, gender			0.7
Hirooka [43]	97 adults ≥18 years 41 % men	CS	Sitting/lying time TV/computer time	Total time in exercise, localization (Japan vs. USA)			0.8
Ishii [63]	1034 adults 40–69 years 52 % men	CS	Time spent in TVSE	Age, gender, education, household income, employment status BMI		Living arrangements, marital status	0.90
Jans [74]	7720 adults Mean age = 32 (SD = 11) 60 % men	CS	Total sedentary time Total sitting time Sitting time at work Sitting time commuting Sitting time during house work Sitting time during the day/evening	Occupational groups, business sectors			0.72
Kaufman [33]	> 20 years	CS	Time spent sedentary outside of work	Smoking			0.86
Kozo [59]	2196 adults Mean age = 45 (SD = 11) 51 % years	CS	Time spent sedentary^O^ Driving/riding in car TV/video viewing Video games Total Sitting minutes Computer/Internet use for leisure Reading Sitting and talking with friends or listening to music Talking on phone	Age, gender, education, income	Neighbourhood walkability index, neighbourhood income	Child living at home	0.90
Kouvonen [51]	38151 adults 17–64 years 20 % men	CS	Time spent sedentary	Work effort-reward balance			0.95
Kozey-Keadle [44]	58 adults 20–60 years 67 % men	QEX	Time spent sedentary	Exercise, intervention to decrease sedentary behaviour			0.64
Lee [85]	410 women age = 42.5 (SD = 9.3)	CS	Time spent sitting in motor vehicles Total sitting time		Pedestrian crossing aids, sidewalk traffic buffers, traffic control device, number of path connections, posted speed limits, neighbourhood attractiveness, neighbourhood safety		0.82
Lepp [46]	302 adults 44 % men	CS	Leisure sedentary activities	Cell phone use			0.82
Li [42]	131 women	CS	Time spent in TVSE	Age, education, work status, lack of PA, BMI, depressive symptoms, Perceived stress, knowledge/beliefs		Marital status, number of children in the household, family functioning	0.95
Mabry [52]	10 adults 50 % men	Q	Barriers to reduce prolonged sitting	Lack of motivation, knowledge/beliefs	Weather, access to facilities	Social norms and community participation	0.80
Menai [41]	2841 adults age 57.3 +/− 5.0 years 38.3 % men	L	Total leisure SB, Leisure TV viewing, leisure computer use, leisure reading, occupational sitting, domestic sitting	PA (leisure, walking, gardening, swimming, biking, occupational, domestic)		Working status: retirement status	0.88
Munir [66]	4436 adults Age from <24 to >55 years 44 % men	CS	Occupational sitting	Age, BMI, PA levels, education, job grade		Married/cohabitating, dependents, work engagement, job demands, job performance	0.84
Oliver [18]	2033 adults 20–65 years 43 % male	CS	Occupational sitting time		Neighbourhood level social deprivation		0.76
Parry [76]	22-59 years	CS	Time spent sedentary^O^			Workdays vs. non-workdays	0.90
Pomerleau [28]	6461 adults 19–65 years	CS	Leisure time spent sedentary	Education, income, smoking, alcohol, vegetables intake	Rural vs. urban setting		0.68
Proper [49]	2650 adults 20–65 years 48 % men	O	Sitting time on weekdays Sitting time on weekend days Sitting in leisure time	Age, gender, education, household income, total PA, working hours	Neighbourhood SES		0.86
Rhodes [72]	206 adults Mean age = 54 (SD = 18.6) 51 % men 174 students Mean age = 22 (SD = 13.2) 26 % men	CS	TV Viewing Computer-Use Reading/Music Socializing	Attitude, intention, perceived behaviour control, subjective norm			0.64
Saidj [17]	2308 adults 18–69 years 46 % men	CS / P	Leisure time sitting		Habitat type (apartment versus house) and habitat size (surface area)	Household size (number of occupants)	0.76
Saidj [53]	35444 adults 44.5 ± 13.0 years 79 % women	CS	Domain-specific sitting time (work, transport, leisure)	Occupation type, perceptions towards PA, age, gender, education		Workdays versus non-workdays	0.84
Salmon [47]	1332 adults > 18 years 45 % men	CS	Time spent sedentary TV Viewing Reading Sitting Socializing	Age, gender, lack of time to be active, enjoyment of PA, preference, tiredness, Injury, disability	Sidewalks, air or noise pollution, weather (perceived as a barrier), safety, no access to facilities	Family commitments, work commitments	0.8
Seguin [25]	92234 women 50–79 years	P	Time spent sedentary	Age, education, ethnicity, perceived health, physical function, previous fall, BMI, chronic diseases, hormone use, medication, alcohol intake, levels of PA, smoking		Marital status	0.8
Stamatakis [80]	7940 adults Mean age = 47 (SD = 18.2) 44 % men	CS	Time spent in TVSE	Education, household income	Neighbourhood deprivation	Social class	0.95
Stamatakis [79]	60404 adults ≥45 years 46 % men	CS	Total sitting time TV viewing Computer time Driving	Education, annual household income	Area-level index of socio-economic advantage		0.95
Stamatakis [22]	2289 adults	CS	TV viewing time Sitting time in work Sitting time outside work	Household income, social class, educational attainment, overall socioeconomic position score	Area deprivation score		0.91
Storgaard [12]	48192 adults 44 % men	CS	Leisure time spent sedentary	Education, employment status	Density of green spaces		0.91
Strong [84]	1374 adults mean age = 45 (SD = 12.9) 25 % men	CS	TV viewing		Neighbourhood problems neighbourhood conditions		0.81
Sugiyama [34]	2224 adults 20–65 years 37 % men	CS	TV Viewing	Age, education working status, income, BMI, leisure time PA	Neighbourhood SES, neighbourhood walkability		0.91
Sugiyama [61]	2046 adults 20–65 years 36 % men	CS	Time spent in other sedentary behaviours (except TV viewing)	Time spent watching TV			0.95
Sugiyama [65]	1408 adults 20–65 years 38 % men	CS	Time spent watching TV	BMI			0.95
Sugiyama [77]	74788 adults >18 years 48 % men	P	Prolonged time in car	Age, work status, household income, car ownership	Suburb, vicinity to CDD	Household composition	0.68
Teychenne [62]	1554 women 18–65 years	CS	TV Viewing	Education, enjoyment of TV, preference for sedentary behaviour, stress and depressive symptoms	Neighbourhood safety, neighbourhood aesthetic, distance to places of interest, distance to physical activity facilities	Social cohesion, social participation, social support	0.92
Thorp [75]	193 adults 34 % men	CS	Time spent sedentary^O^	Type of work		Workdays vs. Non-workdays	0.92
Touvier [78]	1389 adults 45–60 years 50 % men	P	TV Viewing	Retirement			0.95
Uijtdewilligen [27]	11676 adults, women only	P	Time spent sitting at the weekend and time spent sitting on weekdays	BMI, country of birth, highest educational qualification, physical activity levels, smoking, alcohol consumption, stress levels, occupational status	Area of residence	Number of children in the household, marital status, work commitment	0.84
Uijtdewilligen [71]	475 from 13 to 42 years old 47 % men	L	Screen time: TV during leisure on week or weekend days and time spent behind computer during leisure during week and weekend days (h/week)			Daily hassles (like conflicts with colleagues, misbehaving Children and being displeased about personal appearance, and being laughed at,…) Life events (health, work, home/family, personal/social relations, finance)	0.84
Vandelanotte [36]	2532 adults 20–65 years 39 % men	CS	Leisure time internet and computer use	BMI, Other leisure time sedentary behaviour (except TVSE)			0.86
Van Dyck [82]	1200 adults 20–65 years 47 % men	CS	Time spent sedentary^O^	Age, gender, education, employment status, occupation	Neighbourhood walkability index, neighbourhood SES	Living situation	0.95
Van Dyck [55]	419 adults 20–65 years 47 % men	CS	TV Viewing Leisure time internet use	Age, gender, education, employment status, BMI, pros reducing TV viewing, cons reducing TV viewing, self-efficacy reducing TV viewing, pros reducing internet use, cons reducing internet use	Number of PCs, number of TVS, size of the largest TV set	Family social norm TV viewing, friends norm TV viewing, family social norm internet use	0.9
Van Dyck [60]	6014 adults 20–65 years 44 % men	CS	Overall sitting time Motorized transport time	Age, gender, education, having a drivers licence, BMI	Not many cul-de-sacs, not many barriers in neighbourhood, aesthetics, street, connectivity, walking and cycling facilities, access to services, proximity to destinations, number of different type of destinations within 20 min walk from home, parking difficult near local shopping area, traffic safety, crime safety, residential density	Living with a partner	0.95
Van Holle [16]	2839 adults 55–65 years 52 % men	CS	Sitting time during the weekend days		Social trust and cohesion, personal safety, aesthetics, mean destination score, number of TVs in the house	Social participation, social support from friends or colleagues (	0.80
Van Uffelen [24]	8920 women 25–30 years 11018 women 50–55 years	CS	Sitting time	Education, income, studying, occupation, country of birth, alcohol intake, levels of PA, passive leisure activities, poor sleeping, smoking, BMI, chronic conditions, stiff/painful joints	Area of residence	Marital status, number of children, caring for family members	0.90
Wallmann-Sperlich [10]	2000 adults Mean age = 49,3 (SD = 17,6) 48 % men	CS	Sitting time	Age, gender, education, income	Type of residence, aesthetics, access to park and recreational facilities, distance to local facilities, public transport infrastructure, neighbourhood safety -traffic and crime		0.90
Wilson [37]	68 adults 47 % men	CS	Time spent sedentary^O^ TV Viewing	Age, education, family income, employment type, levels of PA, anthropometrics			0.41
Xie [23]	3016 adults ≥18 years 46 % men	CS	TV Viewing	Age, gender, employment, education, BMI, smoking, alcohol intake, vigorous PA		Marital status	0.95
Zolnk [15]	2943 households 25–65 years	CS	Private vehicle commuting time	Income, occupation, gender	Degree of centredness (urban/rural subway)		0.68

*BMI* body mass index, *CBD* central business district

Study design: *CS* cross sectional, *L* longitudinal, *O* observational, *P* prospective, *Q* qualitative, *QEX* quasi-experimental

^a^only study to investigate policy factors: worksite physical activity policy, work place health promotion programme

### Risk of bias

The quality scores for the included 74 studies, expressed as a percentage (with 0 % the worst and 100 % the best possible quality), ranged from 41 to 95 % as illustrated in Table 1. Overall the studies were of good quality with a median score of 85 %. Of all the items on the checklist for the quantitative study quality assessment, items 1 ‘question/objective sufficiently described’, item 2 ‘study design evident and appropriate’ and item 10 ‘analytic methods described/justified and appropriate’ were the most frequently reported. Item 11 ‘some estimate of variance is reported for the main results’ appeared to be the item most frequently missing.

### Measurement of sedentary behaviours

In total, 16 studies objectively measured sedentary time with fifteen using accelerometry (ActiGraph *n* = 14 and activPAL *n* = 1) and one using heart rate. Seven studies used both self-report and objective measures, and the remainder relied upon self-reported sedentary time measurement (*n* = 58). Five domains of self-report sedentariness have been identified (some studies report more than one domain):

1. Total screen time
2. Television and screen entertainment (TVSE)
3. Transport sitting time
4. Total sitting time (including occupational sitting)
5. Leisure sitting time (time outside of work, TVSE, reading/listening to music/socialising)

### Individual correlates

#### Type of individual-level factors

Of the 74 studies included, 62 examined the relationship between sedentary behaviours and individual factors. Four categories of factors were identified: behavioural (lifestyle, physical activity and sedentary habits (*n* = 30)), physical, biological and genetic (age, gender, body composition, health status and medication (*n* = 26)), psychological (stress and depressive symptoms, attitudes and perceptions (*n* = 25)) and socioeconomic status (educational levels, employment/occupational status and income (*n* = 23)). All individual factors were assessed using self-report questionnaires apart from some of the physical, biological and genetic factors (e.g., body mass index and heritability) that were measured objectively. Table 2 provides a detailed overview.

**Table 2:** Individual correlates of sedentary behaviours in adults

Individual Correlates of Sedentary Behaviour in Adults (18–65 years)						
Factors (*n* = total studies)	Total screen time	Leisure screen time	Transport sitting time	Total sitting time	Leisure sitting time	Total Objective SB
Behavioural						
Alcohol consumption (*n* = 5)		nr [23]	+ [26]	+ [27]^W^ nr [24]^W^, [25]^W^		
Alcohol and diet (*n* = 1)					+ [28]	
Food cravings (*n* = 1)				+ [30]^W^		+ [30]^W^
High calorie snacking (*n* = 4)		+ [31], [32], [40]	+[26]			
Lifestyle (*n* = 1)		+ [29]				
Smoking (*n* = 7)		+ [33] nr [23]	+ [26]	+ [24]^W^, [25]^W^, [27]^W^	+ [28]	
Lack of PA (*n* = 1)		nr [42]^W^				
PA (vigorous) (*n* = 2)		nr [23]				nr [44]
PA levels (*n* = 11)	nr [41]	- [31]^W^, [34]^W^, [35]^M^, [36] nr [73], [41]		+ [40] - [24]^W^, [25]^W^ [37], [27]^W^	nr [41]	- [37]
PA outside work (*n* = 2)		- [34]^W^, [39]				- [39]
Total time in exercise (*n* = 1)				nr [43]^M^		
Poor sleeping habits (*n* = 2)			+[26]	- [24]^W^		
Sedentary habits (*n* = 2)		+ [36]		+ [45]		+ [45]
Cell phone use (*n* = 1)		+ [46]				
TV viewing time (*n* = 1)				+ [61]		
Physical/Biological/Genetic						
Age (*n* = 20)	nr [53]	+ [23], [32], [63], [55], [48], [48], [49]^W^, [34]^W^ nr [59], [42]^W^, [9], [31]^W^, [34]^M^	+ [48]	+ [30]^W^, [49]^W^, [50], [10], [60] nr [25]^W^, [47], [37],	+ [53]	+ [30]^W^ nr [37]
Gender (*n* = 19)		- [23], [48] nr [55], [57]	+ [53], [15], [59], [60]	+ [54]^M occ^ - [47], [50], [49], [51], [43], [52] nr [33], [12]	+ [53]	- [37] nr [45], [56]
BMI (*n* = 25)		+ [23], [55], [29], [61], [63], [31]^W^, [49], [34], [35]^M^, [32], [65]^M^, [36], [62]^W^ nr [42]^W^	+ [48]	+ [25]^W^, [48], [64]^W^, [27]^W^ [54]^occ^, [40]^occ^ nr [30]^W^, [24]^W^, [37], [66]^occ^		+ [56] nr [30]^W^, [37]
Chronic diseases (*n* = 4)				+ [25]^W^, [32], [67]^M^ nr [24]^W^		
Disability, Illness, Injury (*n* = 5)			+ [26]	nr [47], nr [30]^W^, [24]^W^, [68]^W^		nr [30]^W^
Hormone use (*n* = 1)				+ [25]^W^		
Medication (*n* = 1)				+ [25]^W^		
Pregnancy (*n* = 1)						+ [70]
Race (*n* = 3)		+ [31]^W^ nr [42]^W^		+ [25]^W^		
Heritability (*n* = 1)						+ [69]
Psychological						
Attitude (*n* = 1)				- [72]		
Depressive symptoms, anxiety, tension or stress (*n* = 7)		+ [42]^W^, [57], [31]^W^ [29] nr [62]^W (med)^	+ [26]	+ [27]^W^		
Enjoyment of TV (*n* = 1)		+ [62]^W^				
Intention (*n* = 3)				-[45], [72] nr [54]^occ^		- [45]
Perceived behavioural control (*n* = 2)				nr [72], [54]^occ^		
Perceived health (*n* = 3)		- [31]^W^		- [25]^W^	nr [53]	
Perceived benefits of reducing SB (*n* = 2)		- [55]		- [52]		
Preference (*n* = 2)		nr [62]^W (med)^		+ [47]		
Subjective Norm (*n* = 2)				+ [52] nr [72]		
Socio-economic Status						
Level of educational attainment (*n* = 22)		- [23], [31]^W^, [42]^W^, [55], [9], [48], [40], [22] nr [63]	nr [48]	+ [10],[53]^occ^, [54]^occ^, [40]^occ^, [22]^occ^ - [24]^W^, [79], [25]^W^, [27]^W^ nr [68]^W^, [66]^occ^	- [28]^M^, [12]	+ [22]
Employed (*n* = 7)		- [23], [31]^W^, [63], [55], [9], [48]			+ [12]	
Manual Employment (*n* = 4)		+ [73]		+ [38] - [37]		+ [22] - [37]
Office work (*n* = 9)		- [40], [54], [22]	+ [46]	+ [74], [38], [27]^W^, [40]^occ^, [53, 54]^occ^, [22]^occ^ -[60], [67]^M^		+ [22], [75]
Work vs non-work time (*n* = 5)			+ [77]	S [74]		S [39], [76], [75]
Full time versus part-time work (*n* = 3)			S [27], [66], [74]	S [27], [66], [74]		
Change at work (*n* = 1)				S [68]		
Work commitment (*n* = 3)				- [67]^M^, [52]	- [47]	
Retirement (*n* = 3)		+ [41, 78], [73]			+ [41]	
Studying (*n* = 1)				+ [24]^W^		
Household Income (*n* = 10)		- [48], [22], [40] nr [63]	+ [77]	+ [59], [80], [22]^occ^, [40]^occ^ - [79]	- [28]^M^	+ [59], [22]
Income (*n* = 8)		- [30]^W^, [73] nr [9]		+ [54]^occ^ - [24]^W^, [30]^W^, [37] nr [10]		- [30]^W,^ [37]

Note: Each result is reported as positive (+), negative (−), or not related (nr) for objective or self-reported/perceived individual measure. Significant associations only in subgroups are identified as men (^M^), women (^w^) ^occ^ refers to occupational time. S refers to significant differences between groups. For one study [62], the studied factor was investigated as a mediator of the association between education and sedentary behaviour and identified as (^med^)

#### Behavioural factors

Thirty studies examined lifestyle factors: alcohol consumption (*n* = 7), food intake (*n* = 5), smoking (*n* = 7) and physical activity (*n* = 17). Alcohol consumption was found to be unrelated to sedentary behaviours in the three of the five studies [23–25] that examined its correlation as an individual factor. The remaining two found it to be positively associated with time spent sedentary in transport (driving) [26] and to overall weekend sedentary time [27]. Similarly when combined with diet, it was shown to have a positive association [28, 29] to TVSE and overall leisure sitting time. Four studies investigating food cravings, snacking and high calorie snacking found that sedentariness was highly associated with all four [27, 30–32]. In six of the seven studies investigating smoking, it was shown to be positively associated with sedentary time as measured by TVSE, time spent driving and total sitting time [24–29, 33]. In terms of physical activity, 18 studies examined its relationship with sedentary behaviour. The majority looked at overall physical activity levels (*n* = 11) and found there to be an inverse association [24, 25, 31, 34–38]. This was also the case for the three studies that explored levels of physical activity outside of work-time [34, 39, 40]. One study examined the association between retirement and physical activity levels in a number of different sedentary domains (TVSE, leisure reading, occupational sitting and domestic sitting) and reported no correlation between it and any of the domains [41]. Similarly, a lack of physical activity [42], vigorous physical activity [23] and total time exercising [43] were not significantly associated with sedentariness. One study conducted a four-armed randomised trial investigating whether (i) supervised exercise, (ii) supervised exercise with advice to decrease sedentary time or (iii) advice to decrease sedentary time and increase non-exercising physical activity levels [44] would change total sedentary time, as measured by an inclinometer. Results revealed that structured exercise was ineffective; only those in the group that were given advice to try and change their sedentary behaviours and increased their daily physical activity levels showed a significant change in total sedentary time. Finally, sedentary habits such as TV viewing and cell phone use (gaming and suing wifi) were found to be positively associated with total time spent sitting [24, 34, 45] and TVSE [36, 46].

#### Physical, biological and genetic factors

Twenty-six studies investigated physical, biological or genetic factors with all of them evaluating age as a correlate of sedentary behaviour. Fourteen of twenty studies supported a positive relationship between age and sedentary behaviours (the older the person, the more sedentary). Eight studies looked at total sitting time, five of which positively correlated with age. Overall the results were mixed between positive correlations and no significant correlations. No studies reported a negative correlation and furthermore findings could not be differentiated by their sedentary measurements.

Gender was investigated in 19 studies. Ten reported the female gender to be inversely related to sedentariness [23, 37, 43, 47–53] with two reporting males to be more sedentary when associated with total time spent in front of a computer screen and overall leisure sitting time [53, 54]. The remainder found little or no association with either gender [12, 33, 45, 55–57]. The majority of studies reported gender differences defined sedentariness as total sitting time [43, 47, 49–51] or TVSE [23, 48] with one using accelerometry [37], one using heart rate [56] and one reporting barriers to changing sedentary time in Oman via semi-structured interviews [52]. Four studies reported the male gender to be positively associated with sitting time in transport [53, 58–60].

The relationship between sedentary behaviour and body mass index (BMI) was evaluated in 25 papers, the majority of which investigated its association with leisure screen time. Seventeen of these studies [23, 25, 29, 31, 32, 34–36, 49, 55, 56, 60–65] reported a positive relationship with the remainder showing no correlation. Nine studies examined this association using total sitting time and two used accelerometers as an objective measure [30, 37] and one heart rate [56]. The two studies that used accelerometry reported no significant relationship between BMI and total sedentary time while the majority of the remaining studies showed that the higher the BMI the higher the level of sedentariness. Two studies looked at the association between occupational sitting and BMI and reported a positive association [54, 66]. Overall results suggest that there is a strong relationship between increased BMI and higher level of sedentary behaviours.

Chronic diseases (e.g., diabetes, cardiovascular disease) were shown to have a positive relationship with sedentary time in three of the four studies [25, 32, 67] that included them. In none of the studies were they the primary factors being investigated. In contrast, illness, previous surgery, disability and injury were shown to have no significant correlation to total sitting time [24, 30, 47, 68]. One study investigated the role of heritability [69], one pregnancy [70], and another the role of hormone treatment and medication [25]. All three reported significant correlations with sedentariness.

#### Psychological factors

Fifteen studies included psychological factors. However few investigated more than one factor. Five studies investigated depressive symptoms and four found that symptoms of depression, anxiety and tension were positively related to total screen time [29, 31, 42, 57]. Similarly perceived stress levels [26, 27, 42] and perceived tiredness [47] were also positively associated, whereas perceived health [25, 31] and perceived benefits of reducing sedentary behaviours [53–55] were found to be inversely associated with sedentariness as measured by occupational sitting, TVSE and total sitting time. One study investigating perception of personal appearance and content with body image found no relationship to sedentariness [71]. Rhodes et al. [72] investigated whether planned behaviour is related to sedentary behaviours. They found mixed results; attitude and intention were negatively correlated with sedentariness as measured by total sitting time while perceived control and norm were not. Conroy et al. [45] also reported that intention and habit in terms of regulating sedentary behaviour were negatively associated. Overall the limited available evidence is supportive for a positive relationship between perceived feelings of depression, stress and anxiety and TVSE and a negative relationship between sedentary behaviour and planned behaviour to overcome sedentariness.

#### Socio-economic factors

Twenty-two studies investigated educational levels and their relationship to sedentary behaviours. Nine examined TVSE, eleven used total sitting time or occupational sitting, two total leisure sitting time and one accelerometry for total sedentary time [22]. Of the nine that examined the correlation between educational levels and TVSE, eight reported significant inverse correlations [9, 22, 23, 31, 40, 42, 48, 55] and one reported no significant relationship [63]. In terms of total sitting time, five studies focused on occupational sitting as a domain of total sitting found there was a positive relationship with educational attainment [10, 22, 40, 53, 54] whereas the studies that investigated total sitting time without classifying domains found it to be negatively correlated. The exception was total sitting time as measured by actigraphy; it was found to have a positive association with educational attainment [22].

Occupation and employment were explored as a potential factor in seventeen studies. In relation to TVSE, it was positively related to unemployment while the opposite was true for employment (negatively correlated) [9, 12, 23, 31, 48, 55, 63]. Storgaard et al. [12] investigated employment in relation to all leisure sitting time as opposed to just TVSE and reported it to be similar to TVSE; positively related to unemployment and negatively related to employment.

Type of employment was reported in some of the studies. Manual employment, investigated in four studies [22, 37, 38, 73] was positively correlated to sedentariness outside of work where sedentariness was measured as total sitting time both subjectively and objectively. In contrast, working in an office was more likely to result in less sedentary time outside of work. Chau et al. [38] and Jans et al. [74] reported increased total sitting time associated with working in an office. Thorp et al. [75] found call centre employees to be more sedentary during the working day than customer service workers. Five further studies exploring type of occupation showed that those in professional roles were more likely to have a higher level of occupation sitting than those in non-professional positions [22, 27, 40, 53, 54].

Five studies focused on whether sedentary behavior differed based on work and non-work time days. Overall, work days corresponded to more sedentary time [39, 74–77] and a greater amount of time spent in prolonged sitting, when compared to non-work days [75, 76]. Saidj et al. [53] reported the more sedentary the occupation type the higher the association with increased sedentary time in other domains (work, transport, leisure, screen time) during weekdays but not during the weekend.

Total sitting time, sitting time during work and traveling to and from work was significantly higher for full-time workers than for part-time workers [27, 66, 74]. Also, work commitments as barriers for physical activity were inversely associated with reported time spent sedentary [47, 52, 67]. Munir reported that vigor at work was associated with less occupational sitting in men and women, but the association was unclear across gender for absorption at work, dedication or job performance [66]. In women, change at work, return to study or new work was associated with an increase in total sitting [68].

Other factors relating to employment that were investigated were retirement and studying. From the retirement perspective, four studies found that retirement resulted in an increase in sedentary behaviour [41, 68, 73, 78]. Retirement was associated with an increase in total [68, 78], leisure SB (screen, reading, total) and domestic sitting [41, 73]. Van Uffelen et al. [24] reported a significant increase in overall sitting time in women who were studying.

Sixteen studies examined the relationship between income and sedentary behaviours using various measures of sedentariness. One study reported a positive association with sitting time spent in transport [77] and three studies that focused on occupational sitting time [22, 40, 54] also found a positive relationship. Of the remaining studies, TVSE as a sedentary measure was used in seven studies, five of which reported a negative relationship [22, 30, 40, 48, 73] while the remaining two showed no correlation [9, 63]. Six studies examined income and its relationship to total sitting time [10, 24, 30, 37, 59, 79, 80]. Finally one study focused on leisure sitting time [28] and found it to be inversely related. In terms of total sitting time and its relationship to income, all studies but one [10] reported a relationship, mainly in a positive direction. Finally in terms of objectively measured total sedentary time and its relationship with household income, both Kozo et al. [59] and Stamatakis et al. [22] found a clear positively association.

### Interpersonal correlates

#### Type of interpersonal level factors

The relationship between interpersonal factors and sedentary behaviours was examined in 22 studies, 14 were cross-sectional; 3 longitudinal, 3 prospective and two were qualitative. Two domains of interpersonal factors were identified, family-related (marital status, living arrangements, family functioning, number of children, family commitment (*n* = 17)) and social factors (social norms, social interaction, cohesion, support and participation, sense of community; (*n* = 7)). All interpersonal factors were assessed using self-administered or interview-administered validated questionnaires. Table 3 provides detailed findings relating to interpersonal factors from the 22 identified papers.

**Table 3:** Interpersonal correlates of sedentary behaviour in adults

Interpersonal correlates of Sedentary Behaviours in Adults (18–65 years)						
Factors (*n* = total studies)	Total screen time	Leisure screen time	Transport sitting time	Total sitting time	Leisure sitting time	Total Objective SB
Family						
Marital status (*n* = 8)		+ [23] - [63] nr [42]^w^	nr [60]	- [24]^w^, [60] nr [27], [68], [66]^occ^		
Living arrangements (*n* = 3)		nr [63], [9] S [48]				
Family functioning (*n* = 1)		- [42]^w^				
Number of children (*n* = 8)		^−^ + [59], [53] nr [9]^w^, [42]^W^	+ [77], [59]	- [24]^w^ [59], [71], [68]^b^	- [59]	- [59]
Family commitment (*n* = 5)				- [24]^w^, [66]^occ^ + [67]^M^, [52]	+ [47]	
Social factors						
Social norms (*n* = 3)		+ [55]		- [52]	nr [16]	
Social cohesion, interaction, support and participation (*n* = 5)		- [62]^med^ nr [9], [62]^med^, [84], [71]			nr [16]	
Sense of community (*n* = 2)		nr [9]		- [52]		

Note: Each result is reported as positive (+), negative (−), or not related (nr) for objective or self-reported/perceived intrapersonal measure. Significant associations only in subgroups are identified as men (^M^), women (^w^). ^f^ refers to friends/colleagues support; ^b^ refer to birth of child; ^occ^ refers to occupational timerefers to occupational S refers to significant differences between groups. For one study [62], the studied factor was investigated as a mediator of the association between education and sedentary behaviour and identified as (^med^)

#### Family-related factors

Eight studies investigated the relationship between “marital status" and sedentary behaviour. In Japanese adults, Ishii et al. [63] reported that unmarried subjects were likely (odds ratio [OR], 2.02; 95 % CI, 1.32–3.10) to spend more time in TVSE than married subjects (>14 h/week). Van Uffelen et al. [24] found sitting time to be significantly higher in single women [24] while van Dyck et al. [60] showed adults living with a partner [60] sat less. Another study conducted in Hong Kong reported contradictory findings. Xie et al. found TV viewing time to be higher in married persons [23].

No relationship was found between TV viewing and marital status in a group of low-income women [42], neither between occupational sitting and marital status in men or women [66]. Clark et al. [68] using a prospective study design investigated the relationship between life events and sedentary behaviour and found change in marital status was not associated with changes in total sitting [68].

In terms of “living arrangement”, whether someone lived alone or with others was not associated with screen time [63] or TV viewing [9]. One study did however report that men living alone were more likely to watch TV for 4 h/day or more [48]. Only one study investigated the association between TV viewing time and lower “family functioning” (likert score) in a group of low-income women and found a positive correlation (*r* = 0.28, *p* < 0.01) that remained significant in a multivariable model including stress and depressive symptoms [42]. The impact of the “number of children” on sedentary behaviour as assessed by TV viewing time was not significant in Li’s study in low-income women [42], nor was it significant in a longitudinal study by Ding [9]. In contrast, several authors found that that overall sitting time was lower with more children [24] or with the birth of child [24, 53, 68, 71]. Kozo et al. investigated several sub-types of sedentary behaviour and found that having no children was related to more TV/video viewing, computer/Internet use, sitting and talking with friends or listening to music, total sitting time or accelerometer-measured sedentary time [59]. Two studies found a positive association between number of children and transport sitting time [59, 77]. Family commitment defined as providing care for other members of the family was investigated in the Australian Longitudinal Study on Women’s Health [24]. It revealed that women who cared for others spent less time sitting, particularly younger women. Similarly, having more dependents was associated with decreased occupational sitting time in men and women [66]. In contrast, Salmon et al. reported family commitments as a factor that decreased physical activity resulting in an increase in total sedentary time [47]. This finding was further supported by George et al. [67] and Mabry et al. [52] in their qualitative studies. They both reported family commitments as a barrier to decreasing sedentary time. Taken together, these results show that family-related factors show inconsistencies for their relationship with sedentariness.

#### Social factors

Six studies investigated other social factors such as social norms [52, 55], social cohesion, interaction, support and participation and sense of community [9, 16, 52, 62]. Social norms were found to correlate with leisure screen time in one study. Although other factors were not significantly associated with sedentary behaviour they were significant mediators of the impact of education on this unhealthy behaviour [62]. No social factors were found to correlate with weekend sitting time with or without interaction with retirement status [16] Finally a study by Uijtdewilligen et al. [27] investigating daily hassles found no significant association with screen time [71].

### Environmental correlates

#### Type of environmental level factors

Of the 74 studies included, 33 considered environmental exposure. Environmental exposures/resources is categorised under five domains, four of which are previously proposed in other publications [81]; physical environment, services available in the environment, socio-demographic environment, neighbourhood safety and the additional domain of the home/work indoor environment. Twenty four studies account for factors related to the physical environment, fourteen thirteen considered variables of the socio-demographic environment (neighbourhood socio-economic status, deprivation), nine examined factors relating to neighbourhood safety and eight investigated service environment variables (recreation facilities, access to services, proximity of destinations). Finally, four studies considered the indoor environment at home or at work (furniture, number of TVs/PCs).

In term of measurement, self-reported/perceived and objective assessments of environmental characteristics were quite equally distributed across the studies. The most commonly used objective measure of environmental factors was GIS techniques and composite environmental measures (e.g., neighbourhood walkability index, neighbourhood deprivation index). Table 4 provides a detailed account of the included studies, the investigated environmental variables and measurement tools.

**Table 4:** Environmental correlates of sedentary behaviours in adults

Environmental Correlates of Sedentary Behaviours in Adults (18–65 years)						
Factors (*n* = total studies)	Total screen time	Leisure screen time	Transport sitting time	Total sitting time	Leisure sitting time	Total Objective SB
Home/work indoor environment						
Number of PCs at home (*n* = 1)		+ [55]				
Number of TVs at home (*n* = 2)		nr [55]			nr [16]	
Size of the largest TV set (*n* = 1)		+ [55]				
Shower facilities at work (*n* = 1)						+ [86]^b^
Lockers for clothes at work (*n* = 1)						+ [86]^b^
Safe bike storage at work (*n* = 1)						+ [86]^b^
Habitat surface area (*n* = 1)					- [17] ns [17]	
Habitat type (apartment vs. house) (*n* = 1)					ns [17]	
Physical environment						
Type of residence (*n* = 1)				nr [10]		
Not many cul-de-sacs/barriers in neighbourhood (*n* = 1)			+ [60]	nr [60]		
Aesthetics/attractiveness (*n* = 6)		nr [9], [62]^W (med)^	+ [85]^W^ nr [60]^a^	- [60]^a^ nr [10]	nr [16]	
Proximity/density of green spaces (*n* = 2)				- [12]^O^, [14]^O^		
Neighbourhood walkability (*n* = 5)		- [34]^W^ ^a^ ^O^, [59]^a^ ^O^, [9] nwr^a^ ^O^	- [59]^a^ ^O^	+ [82]^a^ ^O^ - [59]^a^ ^O^ nr [8]^W^ ^a^ ^O^	nr [59]^a^ ^O^	+ [82]^a^ ^O^ nr [59]^a^ ^O^
Walking and/or cycling facilities (*n* = 4)		- [11] nr [9]	+ [60]^a^	nr [60]^a^, [47]		
Street connectivity (*n* = 2)			- [83]^u O^ nr [60]^a^	nr [60]^a^		
Land –use mix (*n* = 1)			- [83]^u O^			
Traffic safety (*n* = 4)		nr [9], [11]	nr [60]^a^	+[10]^W^ - [60]^W^ ^a^		
Air/noise pollution (*n* = 1)				+ [47]		
Weather as a barrier (*n* = 3)				+ [47], [67], [52]		
Season (*n* = 1)						nr [13]^O^
Living outside State Capital (*n* = 1)		+ [48]^O^				
Living rurally (vs. urban) (*n* = 5)			+[15]^o^, [77]^o^	- [24]^w^, [27]^WO^	+ u[28]	
Region (*n* = 1)						nr [13]^O^
Services available in the environment						
Access to services (*n* = 4)		nr [11]	nr [60]^a^	- [60]^m^ ^a^ nr [47]	nr [16]	
Proximity/distance to destinations (*n* = 3)		nr [62]^W (med)^ ^a^	nr [60]^a^	- [60]^m^ ^a^ nr [10]		
Access to recreation facilities (*n* = 4)		nr [11]		- [52], [67] nr [10]		
Public transport infrastructure (*n* = 2)		- [11]		nr [10]		
Parking difficult near local shopping areas (*n* = 1)			nr [60]	nr [60]		
Socio-demographic environment						
Neighbourhood SES (*n* = 7)		- [8]^W^ ^a^ ^O^, − [34]^w^ ^O^ nr [9]^O^, [59]		+ [79]^a^ + u [49]^a^ ^O^ - u [49]^a^ ^O^ nr [59]^O^	+ [59]^O^ nr [49]^a^ ^O^	+ [59]^O^ nr [82]^O^
Neighbourhood deprivation (*n* = 3)		+ [80]^a^ ^O^, [22]^a^ ^O^		+ [18]^(med)^ nr [22]^a^ ^O occ^		nr [22]^a^ ^O^
Residential density (*n* = 3)		nr [11]	– [83]^O^ nr [60]^a^	+ [60]^a^		
Neighbourhood safety						
Safe park (*n* = 1)		- [11]				
Neighbourhood safety (*n* = 8)		nr [9], [11], [62]^W^ ^a^	+ [85]^W^ nr [60]^a^	- [60]^W^ ^a^ nr [10], [47]	nr [16]	
Neighbourhood problems (*n* = 1)		+ [84]^w^				

Note: Each result is reported as positive (+), negative (−), or not related (nr) for objective or self-reported/perceived environmental measure. Objective measures are identified as (°). Significant associations only in subgroups are identified as men (^M^), women (^w^), non-workers (nwr), and other (u). ^occ^ refers to occupational time. S refers to significant differences between groups. For two studies [18, 62] the studied factor was investigated as a mediator and identified as (^med^). ^a^Composite environmental measure (e.g., neighbourhood deprivation index), ^b^Feature included in a composite environmental measure

#### Physical environment

Mixed results were observed regarding the effect of living in a rural or urban area, dependent on the type of sedentary behaviour considered. Van Uffelen et al. [24] and Uijtdewilligen et al. [27] found that living in an urban area resulted in higher total sitting time among women compared to those living in a rural town. Likewise, Clark et al. [48] reported that living in a regional city outside of the state capitals was associated with an increased likelihood of watching two or more hours of television per day. Pomerleau et al. [28] found similar associations between urban area and sedentary behaviour during leisure time but this was dependent on nationality; Estonian men and Lithuanian women living in towns and cities were more sedentary than their rural counterparts however the opposite is true for Latvian men and women. Two studies showed that living in a rural area was positively associated with transport sitting time [15, 77].

In terms of aesthetics, only one of six studies reported a significant negative association between neighbourhood aesthetics and overall sitting time [60] while five studies reported no associations with sedentary behaviours. Considering green spaces, an increase in the density [12] and a greater proximity [14] were associated with a decrease in sedentary behaviour time. Five studies considered neighbourhood walkability showing mixed results. Three of these reported a negative association with sedentary behaviours. Sugiyama et al. [34] found that women in high-walkability neighbourhoods spend less time watching TV than their counterpart in moderate or low walkability neighbourhoods. Similarly, Kozo et al. [59] showed neighbourhoods with high walkability decrease total sitting time among both men and women. One of the five studies reported an unexpected positive association [82]: the higher the neighbourhood walkability index, the higher the time spent sitting and finally, two studies reported no associations with walkability and total screen time [8] or total sedentary time assessed objectively [59]. Only one of four studies considering walking and cycling facilities found a negative association between bike facilities and sedentary behaviour [11] and no significant associations were found with footpaths. Only one study explored the interaction between work status and neighbourhood walkability reporting a significant correlation [9]. Air or noise pollution and weather were found to be significantly associated with an increase in sedentary time. Findings relating to traffic safety were contradictory [10, 48, 60]. Lastly, no clear patterns of association were observed regarding street connectivity or land mix and sedentary behaviours.

#### Services available in the environment

Regarding the services/destinations resources, only Van Dyck et al. [60] found interactions with gender resulting in significant negative linear relations among men between access, proximity and number of destination close to home and sitting time; whereas six studies found no association with sedentary behaviours. Fields et al. [11] reported a negative association between public transport facilities (i.e., bus stop) and leisure time spent sedentary whereas Wallmann-Sperlich et al. [10] found no significant association with sitting time and public transport facilities.

#### Socio-demographic environment

Neighbourhood socioeconomic status was one of the most commonly investigated environmental factor (*n* = 7 studies) and results are contradictory. Of the seven studies, two studies reported a negative association between neighbourhood socioeconomic status and TV viewing among women [8, 34]. Proper et al. [49] reported greater sitting time for disadvantaged neighbourhoods during weekend days and less sitting time during weekdays while Kozo et al. [59] and Stamatakis et al. [79] found positive associations irrespective of the day of the week. Three studies also reported a positive association between neighbourhood deprivation and leisure screen time and total sitting time [18, 22, 79, 80]. One study reported a significant positive association between residential density and total sitting time [60] whereas results were mixed for time spent sedentary in transport [60, 83] and null for leisure screen time [11].

### Neighbourhood safety

Of eight studies considering neighbourhood/park safety, patterns of associations showed mixed results depending on the type of sedentary behaviour being measured. Fields et al. [11] and van Dyck et al. [60] reported that leisure screen time and total siting time respectively were negatively associated with neighbourhood safety whereas Strong [84] reported a positive correlation for women between neighbourhood problems such as crime and television viewing time. In the opposite, Lee et al. [85] reported a positive association between neighbourhood safety and time spent in car among woman. Seven studies found no association between neighbourhood safety and sedentary behaviours.

#### Home/work indoor environment

Two studies found associations between indoor equipment and sedentary behaviours. Van Dyck et al. [55, 82] found that after adjustment for socio-demographic factors, the size of the largest TV set and the number of computers in the home was positively associated with TV viewing time and leisure-time internet usage. Regarding the indoor work-environment, Crespo et al. [86] found a worksite promotion index including shower facilities at work, lockers for clothes at work and safe bicycle storage to be associated with greater sedentary time, but at the same time also associated with increased levels of recreational physical activity. Finally Saidj et al. found a negative association between habitat surface area and leisure time sitting cross-sectional, while the association was no more significant in a longitudinal perspective [17].

## Discussion

The aim of this review was to summarise the available literature on factors associated with sedentary behaviour in adults aged between 18 and 65 years. We aimed to provide updated information on previously reported factors and identify new ones that should be considered in the development of novel anti-sedentary behaviour interventions for this population. All published literature that met our inclusion criteria was included and themed based on a socio-ecological framework taking into account the different levels of correlates (intrapersonal, interpersonal, environmental and policy) as described by Owens and colleagues [1]. Seventy-four peer reviewed papers focusing on factors that influence sedentary behaviours in healthy adults were analysed, the majority of which were cross- sectional in design, with a significant increase in prospective analyses in the last 2 years. Studies were all conducted across four continents (Australia, Asia, Europe and North America), the majority of which were high-income countries, highlighting the call for studies investigating sedentary behaviours in low or middle-income countries.

Existing literature reports individual correlates such as age, body mass index, physical activity levels, mood and attitude were the most frequently investigated correlates [2, 6]. Rhodes [6] reported that research was very limited in terms of interpersonal and environmental factors that may influence sedentariness in this population [6]. We however have found that recent efforts have focused on these types of correlates specifically environmental factors and there has been an increase in the number of published papers in the past three years reporting these factors and their associations with sedentary behaviours in adults [8–16, 42, 59, 60, 63, 77, 79, 83–85].

In terms of intrapersonal or individual factors, findings were similar to those previously reported demonstrating that those who were more sedentary in terms of either total time or leisure time sedentariness were older, female, did not participate in physical activity or exercise on a regular basis, had a higher body mass index, tended to smoke, consume high calorie snacks and use their cell phone more regularly. Men and especially those with a higher income were more prone to spending time sitting as a means of transport [15, 59, 60, 77].

Very few studies examined psychological factors associated with sedentary behaviour and analyses of those that did were difficult to compare as they all defined the psychological factors being investigated very differently [31, 42, 50, 57, 62]. For example, perceived depression was measured in four studies using four different outcomes measures (two used valid reliable measures while the other asked one question related to feelings of depression and anxiety). The heterogeneity of the factors, population samples and measurement tools used for both the factor and sedentary behaviour makes interpretation and conclusion problematic highlighting the importance of further research into these factors.

The relationship between socioeconomic status (as measured by income, occupation and education) and sedentary behaviour was entirely related to the domain of sedentary time measured; TVSE and educational levels had an inverse relationship whereas self-reported or objectively measured total sedentary time [9, 22–24, 31, 48, 79] was positively correlated. The reason for this discrepancy appears that the higher the education level the more likely one is employed in professional more sedentary roles and this occupational sitting would be included in the measurement of total sedentary time. This emphasises the importance of studies focusing clearly on separate domains of sedentariness. Overall studies showed that socioeconomic status is a significant indicator, perhaps the most consistent factor of all the individual factors identified [9, 23, 31, 48, 60].

Examination of interpersonal factors revealed consistencies throughout. Family related factors were the most frequently investigated, most specifically marital status, cohabiting and number of children in a household. Results were inconsistent highlighting the potential interaction with other individual or environmental factors that may influence the relationship. The influence of other interpersonal factors such as social norms [16, 52, 55] and interaction with friends, peers and colleagues [9, 16, 62, 71, 84] showed no overall relationship to sedentary behaviour. This is an unexpected finding as these factors would be presumed to have a considerable influence on sedentary behaviour as they have been shown to correlate closely with other unhealthy behaviours such as physical activity [87]. Further research into interpersonal factors is required, specifically their interaction with individual and environmental variables.

Of the environmental correlates, the socioeconomic domain was the most common domain studied. Some authors reported a low SES neighbourhood to be associated with an increase in TV viewing time [8, 34, 80] while others found that a high SES neighbourhood resulted in increased sedentary behaviours [49, 59, 79]. More consistently, neighbourhood deprivation was positively associated with leisure screen time [22, 79, 80].

Multiple environmental attributes such as highly walkability neighbourhoods [9, 34, 59, 82], presence of aesthetic features [9, 10, 16, 60, 62, 85], proximity/access to destination and facilities [10, 11, 16, 47, 60, 62], traffic safety [9–11, 60], residential density [11, 60, 83] and a safe environment [9–11, 16, 47, 60, 62, 85] presented numerous inconsistencies in their association with sedentary behaviours, ranging from significant to non-significant to contradictory findings. Multiple environmental factors (i.e., type of residence [10], overall indoor environment [17, 55, 86], type of residence [10], presence of barriers/cul-de-sac [60], street connectivity [60, 83], presence of parking facilities [60], presence of public transportation infrastructure [10, 11] land use mix [83], air/noise pollution [47], and season [13], neighbourhood problems [84] were examined in only one or two studies each, preventing any conclusions being drawn on the significance, the direction or the strength of the association. Thus, further research is required to determine their potential impact on sedentary behaviour. More consistently, presence/proximity of green spaces [12, 14] was repeatedly negatively associated with sedentariness, and living in a rural area was consistently associated with an increase in sitting time in transport [15, 77]. Finally weather was recurrently reported as a barrier and positively associated with total sitting time [47, 52, 67, 88].

The vast majority of studies that used objective environmental measures relied on administrative units or ego-centred definitions of the exposure area (i.e., street network or circular buffers centred on the participants’ activity location) to better evaluate contextual effects on health [89]. Except from one study that examined the indoor worksite environment [86], all studies exclusively assessed the influence of residential neighbourhood on sedentary behaviour and did not account for other geographic life environments. Because individuals are mobile and thus exposed to various non-residential environments during their day-to-day activities [90] accounting solely for the residential environment could misrepresent the relationship between context and sedentary behaviours [91]. Finally, only one study investigated the impact of policy on sedentary behaviour [86]. Interestingly Crespo and colleagues [86] observed that a worksite programme promoting healthy living induced an increase in physical activity levels but at the same time resulted in a parallel increase in sedentary behaviour. Although there is only one study, this may be an important observation in that sedentary behaviour has been recognised as an independent risk factor for disease and should maybe tackled independently from physical inactivity and increasing physical activity may have an adverse effect on sedentariness.

Overall, there seems to be some preliminary evidence for the assertion that environmental characteristics related to both design and recreational resources may explain some of the variance in sedentary behavior. However the variance they explain above and beyond individual and intrapersonal factors remains difficult to ascertain and it is still unclear as to which changes in environments have the ability to affect sedentariness on a relatively permanent basis. Further research is needed to refine hypotheses about how specific environmental variables interact with individual and social factors and how they might be related to particular types and purposes of sedentary behaviour. In addition, policy makers need to begin to consider how to build communities so they facilitate transportation, occupation and recreation options that decrease prolonged sitting time.

With regard to interaction, limited research has been conducted investigating individual, interpersonal and environmental link factors. Kozo et al. [59] identified a relationship between neighbourhood walkability and household income and with each of these two factors independently with sedentary time. Similarly van Dyck et al. [60] reported several associations between different environmental factors, socio-demographic factors and socioeconomic status. Level of educational attainment was found to be inversely associated with income and employment and in turn all three variables were found to be closely related to TVSE by Stamatakis et al. [79] and several other authors. Gender was also shown to be a significant correlate with other intrapersonal factors such as BMI, physical activity levels and lifestyle choices such as smoking and high calorie snacking. Only two studies explored the potential interaction between individual, interpersonal and environmental correlates and age, education, occupation status, working status, retirement position and sedentary behaviours, and found no significant associations [16, 17]. Further analysis is warranted to establish the extent of the influence interactions between the various factors from the different socio-ecological levels have on sedentary behaviour.

Of interest is the finding that there was overall good consistency between self-report measure and objective measures (accelerometry) for most factors but not for all. For example Kozo et al. [59] reported an inverse relationship between neighbourhood walkability and self-report sedentary time but the opposite was true for the relationship between neighbourhood walkability and objectively measured sedentariness. In addition, it is now well acknowledged that the context of the sedentary behaviour is critical to better understand the impact specific factors have on it. As is obvious from the variation of definitions across studies, the notion of sedentariness is plural and refers to different types of sedentary behaviours and correlates [92]. Moreover, though the science around sedentary behaviour is rapidly evolving, some sort of activities (i.e., screen time) that are currently under the sedentary behaviour banner should be reconsidered based on whether they are actually performed in sitting, lying or standing. In order to devise successful interventions to address sedentariness, more detailed and standardised contextual information is essential. In line with Chastin et al. [92], distinguishing between sedentary behaviours by purpose (i.e., work, leisure), environment (i.e., location, type of community, physical environment), type (i.e., screen based or not) and time (i.e., time of the day) would allow researchers to further identify the determinants and correlates of a specific sedentary behaviour and mitigate inconsistencies from previous studies.

The strengths of the review include the large number of abstracts and articles that were screened, the original approach that accounts for levels of correlates based on the socio-ecological model and the discovery of numerous environmental correlates that may need to be considered alongside individual and interpersonal factors when considering interventions to influences sedentary behaviours. Moreover, since the socio-ecological model describes the interrelations between the intra-personal, inter-personal, environmental and policy correlates of health behaviours [1], we also specifically aimed to explore links and interactions between the different levels of correlates.

Several limitations regarding the publication bias and the sampling strategy are acknowledged. Firstly, the paper selection is based on the search terms in the method section which, if absent from the title or the abstract were not detectable and thus relevant studies may have been missed. Secondly, the review is limited to published work, potentially leading to an over representation of significant results and publication bias. Several studies not examining sedentary behaviours as a first outcome and reporting only preliminary analyses are included but where possible, results for adjusted models are reported. In terms of available evidence, the vast majority of studies relied on cross sectional design that limits causal inference and is subject to reverse causality. Consequently the current evidence base is merely about factors associated or correlated with sedentariness rather than determinants or causes of change of sedentary behaviour over time. Finally the heterogeneity of the measurement tools and the plurality of sedentary behaviour definitions make cross study comparison and analyses difficult.

## Conclusions

Despite the fact that numerous questions remain about what determines sedentary behaviour, results from this review provide a plethora of information relating to the multiple factors associates with sedentariness. This information base has increased significantly over the past 5 years since the last published review. It is clear that numerous correlates from individual level through to environmental level factors are important.

More focused research in the future will need to identify the specific settings and type of sedentary behaviour and explore correlates and determinants of setting-specific behaviours.

Longitudinal study designs will allow researchers to identify true determinants and clearly separate them from correlates. Homogeneity in terms of outcome measures would allow more in depth analysis including reporting of effect size and providing more meaningful and useful conclusions. The current evidence base is sufficient to include individual, intrapersonal and environmental factors in the equation when developing and testing interventional designs. However further analysis of interaction between these multiple level factors is essential to optimising any programme or policy focused on disrupting sedentary behaviours and in turn improving population health.

## Additional file

Additional file 1: Table S1. Systematic search terms. **Table S2.** Data Extraction headings. **Table S3.** Checklist for assessing the quality of QUANTITATIVE studies. **Table S4.** Checklist for assessing the quality of QUALITATIVE studies. (PDF 305 kb)
