# Supplementary material for: A systematic review of correlates of sedentary behaviour in adults aged 18–65 years: a socio-ecological approach
Source: BMC Public Health. 2016 Feb 17;16:163. doi: 10.1186/s12889-016-2841-3 (PMC4756464; doi:10.1186/s12889-016-2841-3)
Supplement: Additional file 1: Table S1. — Systematic search terms. Table S2. Data Extraction headings. Table S3. Checklist for assessing the quality of QUANTITATIVE studies. Table S4. Checklist for assessing the quality of QUALITATIVE studies. (PDF 305 kb) [file 12889_2016_2841_MOESM1_ESM.pdf]

Additional Files

Table S1: Systematic Search Terms

**Sedentary behaviour and its synonyms**	("sedentary behaviour" or "sedentary behavior" or "sedentary time" or "sedentariness" or "sedentary lifestyle" or “physical inactivity”)
**Determinants and its synonyms**	(“determinants or correlates or mediators or contributors or association or modifiers or confounders or patterns”)
**Types of sedentary behaviour**	("television watching" or "television viewing" or "video viewing" or "screen time" or "TV" or "video games" or "electronic game playing" or "computer gaming" or "computer time" or "computer use" or "media time" or "media use" or "web browsing" or "screen time" or "bedroom media" or "electronic media" or "PC" or "PC use" or "occupational sitting" or "deskbound" or "reading" or "motor* transport")
**Possible determinants of sedentary behaviour**	(“age or gender or body mass index or weight status or education or income or marital status or ethnicity or "genetic factors" or pain or fatigue or mobility or stiffness or "chronic disease" or “co-morbidities” or “physical function” or disability or impairment)<br>(“motivational or intention or impulsiveness or "cognitive ability" or temperament or attitude or attitudes or "perceived behavioural control" or "perceived physical condition" or planning or "self- efficacy" or "self-esteem" or personality or knowledge or beliefs or "physical activity levels" or "past physical activity levels" or "past sedentary behaviours" or "past sedentary behaviors" or "past sedentariness" or smoking or "family support" or "peer support" or "social support" or "perceived peer attitudes" or "social isolation")<br>(“aesthetics or "accessibility of facilities" or "convenience of facilities" or "availability of recreational space" or "availability of pathways" or "availability of footpaths" or "availability of sidewalks" or "convenience of public transport" or streetlights or "traffic volume" or "traffic safety" or "winter season" or "bad weather" or "air pollution" or "coastal location" or "noise pollution" or "urban sprawl" or "urbanisation" or “walkability” or “environment”)

Table S2: Data Extraction headings

**Heading**	**Data Extracted**
General Information	*title of article, authors, publication year, country where study conducted*
Population Characteristics	*population demographics and baseline characteristics (gender, age range, race/ethnicity, socio-economic status)*
Study Design	*Observational (cross-sectional, longitudinal, prospective, retrospective) experimental or qualitative*
Follow up duration	*Length of follow up and number of follow up points, study completion and attrition rates*
Measures of sedentary behaviour	*Definition of SB, self-report or objective, time of measurement, properties of the measure (reliability and validity where available), domain of SB (leisure, screen, occupational, total sitting time)*
Factors measured	*Determinants, correlates, predictors, mediators*
Statistical Analysis	*Uni-variate, adjusted/multivariate, intention to treat, effect estimates*
Results	*Summary of results – primary and secondary analysis*
Conclusions	*Overview of study findings and recommendations for future research*

Table S3: Checklist for assessing the quality of **QUANTITATIVE** studies

Criteria		Yes (2)	Partial (1)	No (0)	N/A
1	Question / objective sufficiently described?				
2	Study design evident and appropriate?				
3	Method of subject/comparison group selection or source of information/input variables described and appropriate?				
4	Subject and comparison group (if applicable) characteristics sufficiently described?				
5	If interventional and random allocation was possible, was it reported?				
6	If interventional and blinding of investigators was possible, was it reported?				
7	If interventional and blinding of subjects was possible, was it reported?				
8	Outcome and (if applicable) exposure measure(s) well defined and robust to measurement / misclassification bias? Means of assessment reported?				
9	Sample size appropriate?				
10	Analytic methods described/justified and appropriate?				
11	Some estimate of variance is reported for the main results?				
12	Controlling for confounding?				
13	Results reported in sufficient detail?				
14	Conclusion supported by the results?				

SCORING PROCESS

Each question can be answered with “yes”, “partial” or “no”
The summary score is the total sum:

((no of “yes” x2) + (number of “partial” x1) / total possible sum (20) = quality score

Table S4: Checklist for assessing the quality of **QUALITATIVE** studies

	Criteria	Yes (2)	Partial (1)	No (0)
1	Question / objective sufficiently described?			
2	Study design evident and appropriate?			
3	Context for study clear?			
4	Connection to a theoretical framework / wider body of knowledge?			
5	Sampling strategy described, relevant and justified?			
6	Data collection methods clearly described and systematic?			
7	Data analysis clearly described and systematic?			
8	Use of verification procedure(s) to establish credibility?			
9	Conclusion supported by results?			
10	Reflexivity of the account?			

SCORING PROCESS

Each question can be answered with "yes", "partial" or "no"
The summary score is the total sum:

((no of "yes" x2) + (number of "partial" x1) / total possible sum (20) = quality score
